# Ligand-Receptor Interactions and Machine Learning in GCGR and GLP-1R Drug Discovery

**DOI:** 10.3390/ijms22084060

**Published:** 2021-04-14

**Authors:** Mikołaj Mizera, Dorota Latek

**Affiliations:** Faculty of Chemistry, University of Warsaw, Pasteura 1, 02-093 Warsaw, Poland; mikolajmizera@gmail.com

**Keywords:** G protein-coupled receptors, machine learning, gradient boosting, induced-fit docking, virtual screening, molecular docking, scoring functions, drug discovery, glucagon receptor family, GCGR, GLP-1R, secretin receptor family, class B GPCRs

## Abstract

The large amount of data that has been collected so far for G protein-coupled receptors requires machine learning (ML) approaches to fully exploit its potential. Our previous ML model based on gradient boosting used for prediction of drug affinity and selectivity for a receptor subtype was compared with explicit information on ligand-receptor interactions from induced-fit docking. Both methods have proved their usefulness in drug response predictions. Yet, their successful combination still requires allosteric/orthosteric assignment of ligands from datasets. Our ligand datasets included activities of two members of the secretin receptor family: GCGR and GLP-1R. Simultaneous activation of two or three receptors of this family by dual or triple agonists is not a typical kind of information included in compound databases. A precise allosteric/orthosteric ligand assignment requires a continuous update based on new structural and biological data. This data incompleteness remains the main obstacle for current ML methods applied to class B GPCR drug discovery. Even so, for these two class B receptors, our ligand-based ML model demonstrated high accuracy (5-fold cross-validation Q^2^ > 0.63 and Q^2^ > 0.67 for GLP-1R and GCGR, respectively). In addition, we performed a ligand annotation using recent cryogenic-electron microscopy (cryo-EM) and X-ray crystallographic data on small-molecule complexes of GCGR and GLP-1R. As a result, we assigned GLP-1R and GCGR actives deposited in ChEMBL to four small-molecule binding sites occupied by positive and negative allosteric modulators and a full agonist. Annotated compounds were added to our recently released repository of GPCR data.

## 1. Introduction

The glucagon receptor subfamily of secretin-like G protein-coupled receptors includes GCGR, GLP-1R (and GLP-2R), and GIPR. A physiological model of their activation involves peptide agonists: glucagon, GLP-1, GLP-2, and GIP and is involved in G protein-mediated signal transduction that leads to an increase or decrease in glucose/insulin blood serum levels [1,2]. Recent lifestyle and diet changes with increasingly reported drug side effects have brought attention to new antidiabetic pharmacotherapies involving these receptors [3]. Until recently, drug discovery for the glucagon receptor family succeeded in more potent analogs of endogenous peptides targeting the orthosteric binding site and small-molecule ligands targeting the extra-helical, allosteric sites of these receptors [2,4]. The later ones, with a V-shaped conformation, block transmembrane helix 6 (TMH6) deformation and prevent receptor activation. Thus, they act as negative allosteric modulators (NAMs), which binding modes has been discovered only recently [5,6,7]. Although non-peptide agonists of the glucagon receptor subfamily members were known, e.g., Boc5 or BETP, it was not clear how they could interact with transmembrane domains (TMDs) [8,9]. Spacious orthosteric binding sites in all known at that time structures of secretin-like GPCRs (named also class B GPCRs) seemed to be less druggable for small-molecules compounds comparing class A, except for the region deep inside of the receptor core like in, e.g., CRF_1_ (PDB id: 4K5Y) [10,11,12]. Recent advances in cryo-EM have finally provided a detailed structural description of class B GPCR receptors bound to small-molecule agonists [13,14,15,16,17]. Namely, three additional small-molecule binding sites of GLP-1R, located in or close to the TMD orthosteric site, have been revealed, providing a complete picture of class B GPCRs activation [13,14,16,17].

Despite these recent advances in cryo-EM, cheminformatics data for the glucagon receptor family deposited in, e.g., ChEMBL—an open-access bioactivity database [18], is still ambiguous and not mapped into all accessible structural data gathered so far for these receptors. This prevents efficient use of pharmacological data by machine learning algorithms in searching for new or more potent actives. As a result, the performance of ligand-based approaches to drug discovery is still limited and below the actual ML algorithms capabilities.

In this study, we mapped GCGR and GLP-1R actives deposited in ChEMBL onto structural data for these receptors, including their four different small-molecule binding sites. This ligands annotation was not limited to the ortho/allostery distinction, like in a previous study by Burggraaff et al. [19], but also included a distinction between three different allosteric binding sites observed so far in structures of GCGR and GLP-1R receptors [5,6,7,13,15].

In parallel, we demonstrated that both ligand-based and structure-based approaches to GPCR drug discovery are sensitive to subtle differences between compounds targeting different binding sites in the receptor. Only based on results either from ML or from molecular docking we were able to select one distinct group of GLP-1R ligands among all curated GLP-1R ligands derived from ChEMBL. What is more, both approaches, molecular docking, and machine learning, demonstrated moderate-to-high accuracy in the prediction of half-maximal response concentrations of GCGR and GLP-1R compounds. Nevertheless, their efficient combination, like in, for example, 4D-QSAR [20], still requires the precise ligands annotation regarding their binding sites in a receptor. Recently, Venugopal et al. [21] developed a 3D-QSAR model for GCGR antagonists combined with molecular docking and molecular dynamics simulations. The model was developed using the Partial Least Square regression method and 58 structurally similar β-alanine derivatives (44 in a training set and 14 in a test set) that were presumed to bind the extra-helical, allosteric binding site of GCGR. The model demonstrated good statistical characteristics with Q^2^ equal to 0.83, yet for a much smaller dataset (58 vs. 650 compounds) corresponding to a limited subset of the chemical space of GCGR actives (β-alanine derivatives) compared to the ChEMBL dataset.

The main aim of this study was to develop a machine learning approach to identify new actives of the glucagon receptor family. We achieved Q^2^ equal to 0.63 (±0.07) for GLP-1R and 0.67 (±0.04) for GCGR in prediction of half maximal effective/inhibitory concentration (pEC_50_/pIC_50_), respectively. We also attempted ML for GIPR, yet the curated ChEMBL dataset for this receptor was too small for the current study. Our model can be used not only in the prediction of GCGR and GLP-1R actives separately but also in searching for dual-action compounds that could demonstrate increased efficiency in pharmacotherapy without causing additional adverse effects [22]. Known-to-date peptide compounds demonstrating a dual-action effect on glucagon receptor family members include dual agonists of GLP-1R/GIPR [23] and GLP1R/GCGR [24], and triple agonists of GLP-1R/GIPR/GCGR [2]. Previous studies [25,26] also reported peptide compounds that were simultaneously GLP-1R agonists and GCGR antagonists.

In this paper, the approach we used to model the structure-activity relationship for GLP-1R agonism, and GCGR antagonism was based on gradient boosting (GB) and our recent study [27]. The structures of compounds with known pEC_50_ values for GLP-1R and pIC_50_ values for GCGR after curation were parametrized with fingerprints and used for model development and validation.

## 2. Results

### 2.1. Recent Advances in Crystallography and Cryo-EM of the Glucagon Receptor Family

Activation of class B GPCRs involves two-domain interactions with peptide agonists that span across a transmembrane domain (TMD) and an extracellular domain (ECD) of a receptor. It is known, that small-molecule compounds may act as negative allosteric modulators when bound to the extra-helical region of TMD [5,7]. Till recently, there had been no complexes with small-molecule ligands targeting orthosteric sites of class B GPCRs described by either X-ray crystallography or cryo-EM and it was not clear how small-molecule agonists could interact with TMD [10,11]. Based on the first structure of class B GPCR (GCGR) [28], it seemed that most residues important for ligand-based activation of class B GPCRs were rather located further down in the receptor core comparing class A GPCRs [11]. The exception of CRF_1_ [12] with NAM located deeply in the orthosteric pocket and not in a typical orthosteric region observed in class A GPCRs, seemed to confirm this assumption. Nevertheless, early biochemical data on the GLP-1R response to a non-peptide agonist BETP confirmed that it indeed interacts with TMD, yet demonstrating a different binding mode comparing, e.g., a peptide agonist exendin-4 [9].

**Figure 1:**
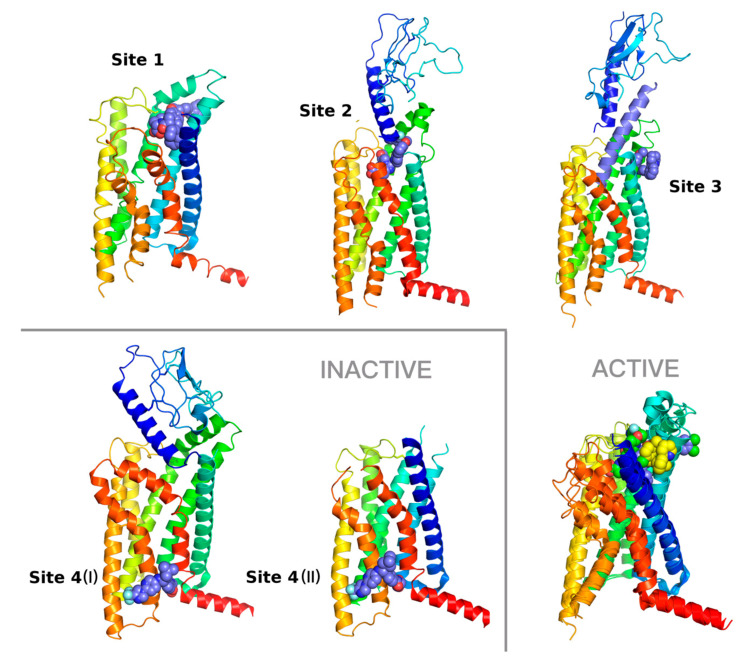
Diversity of small-molecule binding sites in GLP-1R. Binding site 1—6ORV [13], site 2—7C2E [14], site 3—6VCB (Bueno et al. 2020) with an included peptide agonist shown in blue, site 4 (I) (left)—6LN2 (Wu et al. 2020), site 4 (II) (right)—5VEW [15]. Binding sites 1 and 3 corresponds to allosteric sites with positive allosteric modulators (PAMs), site 2 to the orthosteric site with a full agonist. The binding site 4, showed here in 6LN2 (4 (I)) and 5VEW (4 (II)) structures, is occupied by negative allosteric modulators (NAMs). In three cases (2, 3, and 4 (I)), an extracellular domain (ECD) of GLP-1R is also shown. Additionally, an overlay of three active conformations of the GLP-1R receptor with binding sites 1–3 is shown on the right bottom. In this additional superposition of GLP-1R structures the site 1 ligand is shown in blue, the site 2 ligand in green, and the site 3 ligand in yellow. The intracellular part of the receptor that interacts with a G protein complex is nearly identical in all these three superposed structures. However, the extracellular part of GLP-1R differs significantly from binding sites 1 to 3. The most significant differences can be observed in the top region of TMH7 (red), the top region of TMH1 (blue), and in the extracellular loop EC1 region of the receptor that forms a small helix. The structural diversity observed in conformations of EC3 is mostly due to differences in in the top region of TMH7, which is much closer to the ligand-binding sites than TMH6 (orange).

In our recent work [3,29], we hypothesized, based on results from receptor-based virtual screening, that small-molecule ligands could effectively bind also orthosteric sites of class B GPCRs in a similar way to class A GPCRs. Recent advances in cryo-EM of class B GPCRs have proved that orthosteric sites of glucagon receptor family members are indeed druggable for small-molecule ligands [14,16,17]. Small-molecule full agonists of class B GPCRs bind to the orthosteric site of the receptor—the binding site 2 (see Figure 1). What is more, small-molecule allosteric modulators of class B GPCRs can bind not only the extra-helical, membrane-facing, TMD site (the binding site 4, see Figure 1 [5,6,7], but also the interior and exterior of the orthosteric site (the binding site 1 and 3, see Figure 1) [13,15]. However, no structure of a class B member with a small-molecule antagonist (or inverse agonist) located in the orthosteric site, as observed in the case of class A ligands, e.g., carazolol (β_2_AR) [30], has been obtained.

**Figure 2:**
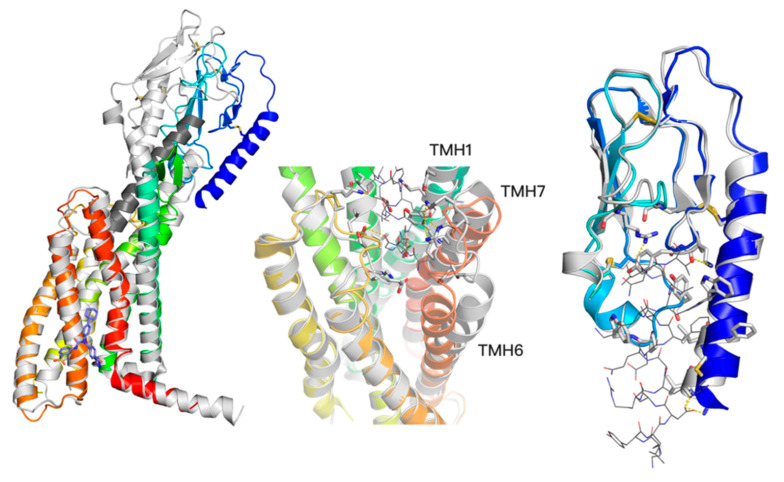
**A** two-domain activation model of GCGR (left) with a comparison between an active conformation (grey, 5QYZ) with a peptide agonist bound (dark grey) and an inactive conformation (blue-to-red, 5XEZ) with a negative allosteric modulator (blue, shown in sticks). The bottom region of TMD changes significantly in the TMH6 region upon the receptor activation (left). The ECD domain (blue) moves closer to the Z axis of the receptor upon the activation (left), yet without significant changes of its conformation (right, superimposed active—grey, and inactive—blue ECD conformations with a truncated (for visibility) peptide agonist from 5YQZ shown in lines). In contrast to slight ECD changes, the top region of TMD domain (middle) noticeably changes upon the peptide agonist binding. Namely, transmembrane helices TMH1, TMH6, and TMH7 move away from the receptor core. The allosteric binding site of NAMs (left) corresponds to the binding site 4 (I) and 4 (II) of GLP-1R ligands shown in Figure 1.

The first structure of a member of the glucagon receptor family was the structure of an inactive conformation of GCGR without any ligand (PDB id: 4L6R) [28]. The next structure released was the GCGR structure (5EE7) [31] that included an allosteric V-shaped modulator located in the extra-helical region of TMD, between TMH6 and TMH7, and facing the lipid bilayer. In the following years, other structures of the glucagon receptor family members have been solved, a semi-active structure (5NX2) [32], an active and peptide agonist-bound structure (5YQZ) [33], and inactive structures bound to allosteric modulators (also V-shaped) [5,6] (see Figure 2). In 2020, new structures of GLP-1R appeared (see Table 1) [13,14,15] that surprisingly showed new small-molecule binding sites in class B receptors (binding sites 1-3, see Figure 1) in the peptide-fitted orthosteric site. Although one of these PDB structures included a rather large, small-molecule full agonist resembling a short, linear peptide (see Table 1, 7C2E), one structure (6VCB) included a much smaller PAM modulator located between TMH1 and TMH2 (see Figure 1).

The most significant structural differences between active and inactive receptor conformations can be observed in the case of 6ORV (Site 1) vs. inactive 6LN2 or 5VEW (see Figure 1). These conformational changes include not only the intracellular part of TMH6, interacting with a G protein complex, but also the extracellular part of the receptor—a movement of TMH6 and TMH5 away from the receptor core upon the ligand binding. The 6ORV ligand is large and partly located below the extracellular loop EC1. The binding site 2 (7C2E) can be also observed in two other PDB entries (see Table 1—6XOX and 6X19) that include ligands dissimilar to the 7C2E ligand. Nevertheless, these 6XOX and 6X19 ligands were also dissimilar (data not shown) regarding Daylight/Tanimoto descriptors to compounds from the ChEMBL dataset, so we discarded these two structures in the study for computational reasons.

Known-to-date structures of GCGR include peptide analogs of glucagon bound to the orthosteric site, and NAMs bound to the allosteric, extra-helical, lipid bilayer-facing site (see Figure 2). Notably, the 5XEZ structure of GCGR includes the same compound as the 5VEX structure of GLP-1R (see Table 1), in both cases bound to the same binding site 4. This proves a frequent lack of selectivity observed between ligands of the glucagon receptor family members and have also been confirmed by recent findings of other dual and triple agonists [2]. Based on this, it is plausible that GCGR (or GIPR) may include the same binding sites 1-4 as observed in all released to date GLP-1R structures.

### 2.2. Annotation of Compounds Deposited in ChEMBL

For 265 GLP-1R ligands and 650 GCGR ligands extracted from ChEMBL, we computed Daylight/Tanimoto descriptors to assess their similarity to PDB ligands shown in Table 1. Among 265 curated ligands of GLP-1R receptor that were extracted from ChEMBL at the time of the current study (September 2020), 230 compounds were similar to the linear-shaped ligand from 6ORV [13]. Both datasets, including 265 and 230 compounds were deposited in our recently released repository (see: https://db-gpcr.chem.uw.edu.pl). The remaining 35 actives were not similar to any of the other known PDB ligands of GLP-1R (see Table 1). In the case of GCGR, the ChEMBL dataset was more diverse. Ninety-four ligands among 650 ligands derived from ChEMBL possessed a distinct V-shape and were similar to the allosteric modulator of GCGR from 5XEZ. 5XEZ includes NAM located in the binding site 4, the only small-molecule binding site in TMD of GCGR that has been deposited in PDB so far. Interestingly, GCGR ligands deposited in ChEMBL were more similar to the 5XEZ ligand than to the best compound from [21]—compound 20 (94 compounds with Daylight/Tanimoto coefficient in the range of 0.577–0.150 vs. the 5XEZ ligand and only 12 compounds in the maximal range of 0.191–0.150 vs. compound 20, respectively).

As we mentioned above, it is plausible that GCGR, as a close homolog of GLP-1R, could also be activated by similar small-molecule orthosteric ligands, like GLP-1R. Unfortunately, the GCGR dataset derived from ChEMBL included only compounds tested for the receptor inhibition, and no ligands similar to the ligand from the binding site 1, 2, or 3 of GLP-1R were included in it.

As a result of the above similarity search and ligand type assignment, we divided ChEMBL-derived compounds into the following subsets (see: https://db-gpcr.chem.uw.edu.pl). In the case of GLP-1R, there were two subsets—compounds similar to the binding site 1 ligand and ‘other’ compounds, not similar to any of the binding site 2, 3, or 4 ligands. In the case of GCGR, there were also two subsets—compounds similar to the binding site 4 ligand extracted from 5XEZ and ‘other’ compounds.

In the case of GLP-1R, compounds similar to the binding site 1 PDB ligand demonstrated better response (high pEC_50_, see Supplementary Figure S1) comparing the ‘other’ subset, which could be the reason why the complex of GLP-1R with the binding site 1 ligand succeeded in structural studies (PDB id: 6ORV). A better response could be related in this case to a higher affinity for GLP-1R and thus possibly to better stability of the ligand-receptor complex. Interestingly, compounds similar to the binding site 1 ligand were also of higher AlogP (high lipophilicity and hydrophobicity and thus high blood-brain-barrier permeability, see Supplementary Figure S2) comparing the ‘other’ subset. On the other hand, GCGR compounds similar to the binding site 4 ligands were of medium and best pIC_50_ values (see Supplementary Figure S1), but they did not form a separate cluster in terms of either pIC_50_, atomic 1-octanol/water partition coefficient AlogP, or Autodock VINA [34] scores like in the case of GLP-1R actives similar to the binding site 1 ligand.

### 2.3. Response to Drug-Receptor Structure-Based Predictions

**Figure 3:**
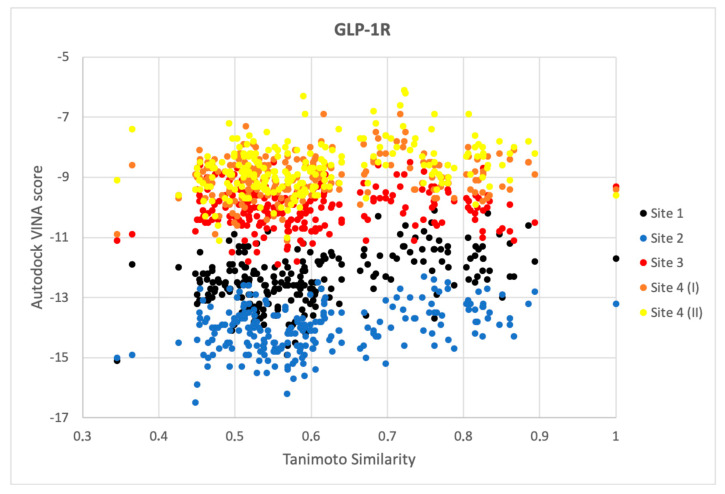
Results for GLP-1R compounds similar to the binding site 1 ligand. The lower binding energy (Autodock VINA score), the better fitness between the ligand and the type of the receptor binding site. Here, sites 1–4 notation corresponds to the notation presented in Figure 1. Site 4 (I) corresponds to the binding site of NAM in 6LN2, site 4 (II)—to the binding site of NAM in 5VEW.

Ligands from the ChEMBL datasets were docked to four different types of binding sites of GLP-1R. As expected, compounds similar to the 6ORV binding site 1 ligand [13] demonstrated the best affinity to GLP-1R structures with binding sites 1 and 2 (see Figure 3). Binding sites 3, 4 (I) (Wu et al., 2020), and 4 (II) were least fitted to ChEMBL-derived compounds, possibly also because they were simply less cavity-like. The PDB ligand from 6ORV (the binding site 1 ligand) that was also found in the ChEMBL dataset was not of the best affinity to GLP-1R and also not the best in terms of pEC_50_ (see Supplementary Figure S1). Among compounds similar to the binding site 1 ligand (see Figure 3), an inversed trend was observed between similarity of compounds to this ligand and predicted binding affinity for the receptor. It suggests that although data on half-maximal response concentrations of compounds were probably useful in discarding them from the ‘other’ subset it may not necessarily provide information which compound is of the best affinity for GLP-1R. It is worth noting that according to the recent study by Nguyen et al. [35] Autodock4 could be slightly more accurate in affinity predictions while Autodock VINA outperforms it in binding mode predictions.

**Figure 4:**
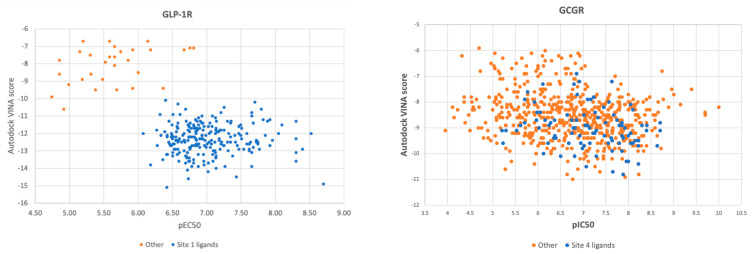
Comparison of Autodock VINA scores obtained from flexible ligand-flexible receptor molecular docking with pEC_50_ (GLP-1R, left) and pIC_50_ (GCGR, right) values.

In Figure 4, we juxtaposed pEC_50_ (pIC_50_ for GCGR) of all ChEMBL-derived compounds with Autodock VINA scores obtained from molecular docking to the binding site 1 (GLP-1R and 6ORV) and to the binding site 4 (GCGR and 5XEZ), respectively. In the case of GLP-1R, results were split into two sets, and pEC_50_ correlated with docking scores. When we examined ligands from the subset of the lowest binding energy and the highest pEC_50_, we found out that this subset included ligands similar to the binding site 1 ligand from 6ORV. These ligands were of high lipophilicity (see the AlogP plot in Supplementary Figure S2). Interestingly, the ‘other’ subset was always predicted as of lower binding affinity regardless of the binding site used in molecular docking (see Supplementary Figure S3). What is more, this set of GLP-1R ligands were examined in functional assays while the ‘other’ subset was mainly from binding assays (see Supplementary Figure S4).

The most important conclusion from this part of the study was that the Autodock VINA scoring function enabled to accurately select compounds similar to the binding site 1 ligand from the ChEMBL dataset. This proves that molecular docking could also be used in ligands annotation along with Daylight/Tanimoto descriptors when such detailed data like the type of the binding site cannot be extracted by a text mining protocol from databases or simply does not exist yet.

In the case of GCGR, the ChEMBL dataset was more evenly distributed. Compounds similar to the binding site 4 ligand did not form a separate cluster in docking results like in the case of GLP-1R compounds. However, these compounds similar to the site 4 ligand were still located in the area of medium-to-best Autodock VINA scores and medium-to-highest pIC_50_ values. Docking results correlated with experimental results (pIC_50_) to a lesser extent than in the case of GLP-1R.

### 2.4. Response to Drug—Ligand-Based Predictions

**Figure 5:**
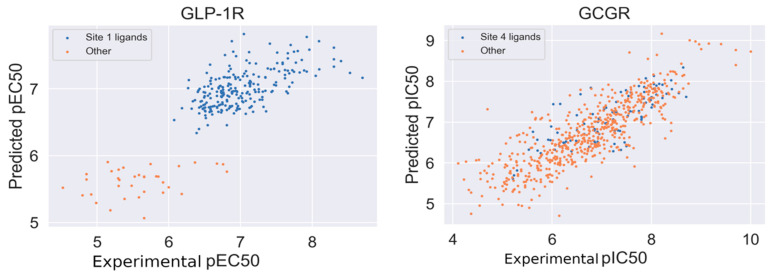
Autocorrelation plots of experimental vs. predicted values of pEC_50_ (GLP-1R) and experimental vs. predicted values of pIC_50_ (GCGR). Correlation coefficient equal to 0.81 (GLP-1R) and 0.83 (GCGR).

We applied the curated compounds data for the development and validation of GLP-1R and GCGR models. The statistical characteristics of the QSAR model for GLP-1R computed in 5-fold cross-validation procedure is Q^2^ = 0.63 (±0.07) and for GCGR is Q^2^ = 0.67 (±0.04). We also computed mean absolute errors for both models. MAE for GCGR was 0.445 (±0.031) and for GLP-1R was 0.32 (±0.028). The autocorrelation plots were presented in Figure 5. Datasets were divided based on similarity to the binding site 1 (GLP-1R) and site 4 (GCGR) ligands. In the case of GLP-1R (left of Figure 5), compounds similar to the site 1 ligand demonstrated a slightly better correlation between predicted and experimental pEC_50_. In Supplementary Figure S4, we presented similar autocorrelation plots but with datasets divided based on the ChEMBL-derived type of assay (‘Binding’ and ‘Functional’). In the case of GLP-1R, compounds similar to the binding site 1 PDB ligand were mostly tested in ‘Binding’ assays while ‘other’ compounds were evaluated using ‘Functional’ assays.

**Figure 6:**
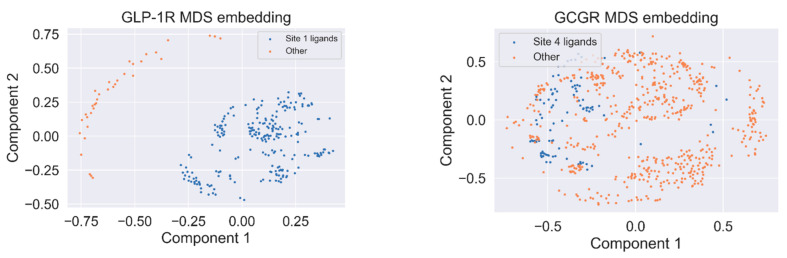
Multidimensional scaling embedding (MDS) for the relative similarity of ligand structures in the GLP-1R dataset (**left**) and the GCGR dataset (**right**).

To investigate the source of the discriminatory effect, we projected fingerprints onto 2D embedding space using multidimensional scaling with Jaccard distance. The embedding is presented in Figure 6. A similar distribution of clusters, as shown in Figure 6, was also visible when using the unsupervised clustering of fingerprints. Compounds similar to the binding site 1 ligand of GLP-1R form the same cluster and, what is more, demonstrate higher pEC_50_ comparing the ‘other’ subset. Our QSAR model reflects these differences in ligand structures and properties between the ‘Site 1’ and ‘other’ subsets.

For GCGR, half-maximal response concentrations were not dependent on the similarity of compounds to the binding site 4 ligands to such an extent as for GLP-1R. We also did not observe such distinct cluster distribution as in the case of GLP-1R.

**Figure 7:**
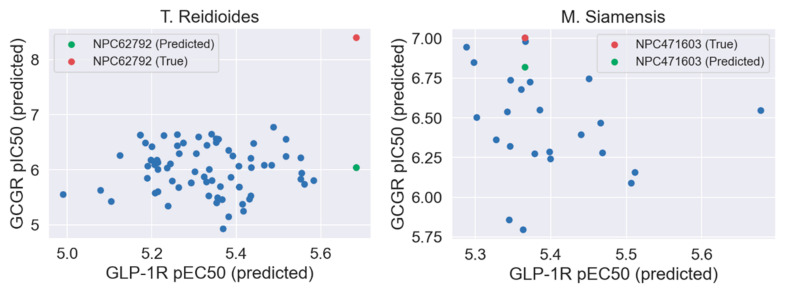
Evaluation of CMAUP-derived compounds using our GCGR and GLP-1R models.

At the last stage of our study, we used our GCGR and GLP-1R models for compounds deposited in CMAUP, a database of Collective Molecular Activities of Useful Plants [36] (see Figure 7). The term ‘glucagon receptor’ provided two species, *Mammea siamensis* and *Trigonostemon reidioides*, with two compounds that demonstrated nanomolar activity for GCGR (see Supplementary Figure S6). NPC471603, a compound found in *M. siamensis* demonstrated the 80 nM activity for GCGR. The better inhibitor activity (4 nM) demonstrated NPC62792 from *T. reidioides*. None of these compounds were similar to any of the PDB ligands targeting the binding sites 1–4 of GLP-1R or site 4 of GCGR. However, based on the shape of these compounds (see Supplementary Figure S6), we suggest that among these two compounds, the *M. siamensis* compound, which has three aromatic groups (phenyl and pyridine) around the imidazole ring, could demonstrate the binding mode similar to the site 4 ligand binding mode.

**Figure 8:**
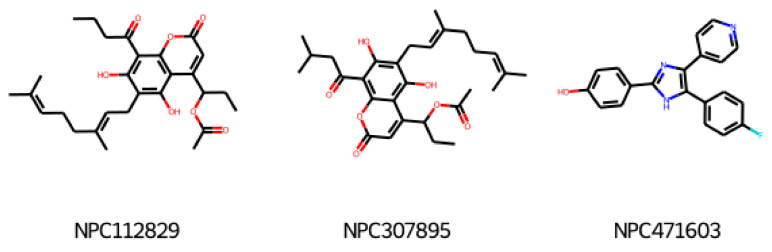
Top compounds found by the GCGR model in the CMAUP subset for *Mammea siamensis*. One of these compounds (right) was also suggested in CMAUP as GCGR active (80 nM).

None of the compounds derived from CMAUP was described as active for GLP-1R. The GLP-1R model that we applied to 24 (*M. siamensis*) and 73 (*T. reidioides*) compounds also did not succeed in finding a potentially active compound (pEC_50_ > 7) for this receptor. As expected, predicted GLP-1R pEC_50_ values for compounds extracted from both of these species did not exceed 6 (Figure 7). Yet, one compound from *M. siamensis* was distinctly better than the others (see Supplementary Figure S7), with predicted pEC_50_ > 5.6. In the case of GCGR, our model predicted higher pIC_50_, around 7, for a few compounds extracted from *M. siamensis*. Among these top compounds (see Figure 8), we found NPC471603 that had experimentally confirmed activity for GCGR (see above). None of the described above five compounds (two for GLP-1R and three for GCGR) were similar to the most potent compound 20 from [21].

## 3. Discussion

ChEMBL datasets for the glucagon receptor family require a more detailed description in terms of allostery/orthostery and in terms of four different binding sites that have been discovered for GLP-1R. Lack of this information severely impedes the performance of both ligand-based and structure-based approaches to drug design. While the ortho/allostery ligand assignment often can be done by a text mining approach [19], extracting information about the specific binding site referring to the current data in PDB (two sites for PAMs, one for NAMs, and one for a full agonist) remains difficult to solve. Without such ligand annotation, ML algorithms are trained on ambiguous data and that decreases their performance in blind test experiments despite the actual great capabilities. As we also demonstrated in this study, molecular docking is sensitive to differences between ortho/allosteric ligands. Yet, an efficient combination of ML and molecular docking in the case of large and diverse datasets requires a thorough ligands annotation to avoid noise in the data that could decrease the ML algorithm performance.

We developed and validated QSAR models applicable to the prediction of pEC_50_ for GLP-1R and pIC_50_ for GCGR. For GLP-1R, both structure-based and ligand-based models were able to discriminate compounds similar to the binding site 1 ligand. Developed QSAR models allow to predict the absolute measure of agonist/antagonist effects on the receptor (pEC_50_ and pIC_50_, respectively) with Q^2^ = 0.63 (±0.07) (GLP-1R) and Q^2^ = 0.67 (±0.04) (GCGR). Although the QSAR model developed for GCGR by Venugopal et al. [21] achieved better statistical characteristics, they were not informative in terms of model robustness in virtual screening for novel chemotypes. Namely, this QSAR model was developed using a small dataset of structurally similar ligands. In contrast, to develop our QSAR model, we used much larger, unrestricted, structurally diverse datasets. Thus, our model applies to a wider domain of chemical structures but at the cost of lower Q^2^ values.

Structural analysis of ligands in datasets showed a relationship between the compound similarity to the binding site 1 ligand of GLP-1R and its high agonist activity. This analysis demonstrated the complexity of the GPCR ligands space in terms of ortho/allostery and possible locations of PAMs/NAMs binding sites. This complexity is weakly reflected in chemical databases and thus impedes the efficient usage of ML algorithms in drug design.

## 4. Materials and Methods

### 4.1. Data Acquisition

PDB ligands (ligands derived from PDB structures of receptors) were extracted and compared to ChEMBL-derived datasets of experimentally confirmed actives of the glucagon receptor family members. GLP-1R and GCGR actives were derived from the ChEMBL database [37]. The agonist activity data against GLP-1R was collected from a dataset with the identifier CHEMBL1784, while the inhibition data against GCGR was collected from the dataset CHEMBL1985. The datasets were downloaded as CSV files and converted into a pandas data frame [38].

### 4.2. Data Analysis

The resulting datasets were joined with metadata regarding the type of source assays (‘Binding’/‘Functional’) that was also acquired from ChEMBL. To assess the similarity of ChEMBL database compounds to PDB ligands from binding sites 1–4 (GLP-1R, see Section 2) and from binding site 4 (GCGR, see Section 2), we used Daylight fingerprints [39] to compute Tanimoto coefficients [40] using the functionality implemented in Maestro [41]. Daylight/Tanimoto similarity descriptors were used for the selection of the most/least similar ligands to the PDB ones. Results of this ligands annotation were included in Supplementary Figures S1 and S2 and deposited in: https://db-gpcr.chem.uw.edu.pl (December 2020).

### 4.3. Data Curation

**Figure 9:**
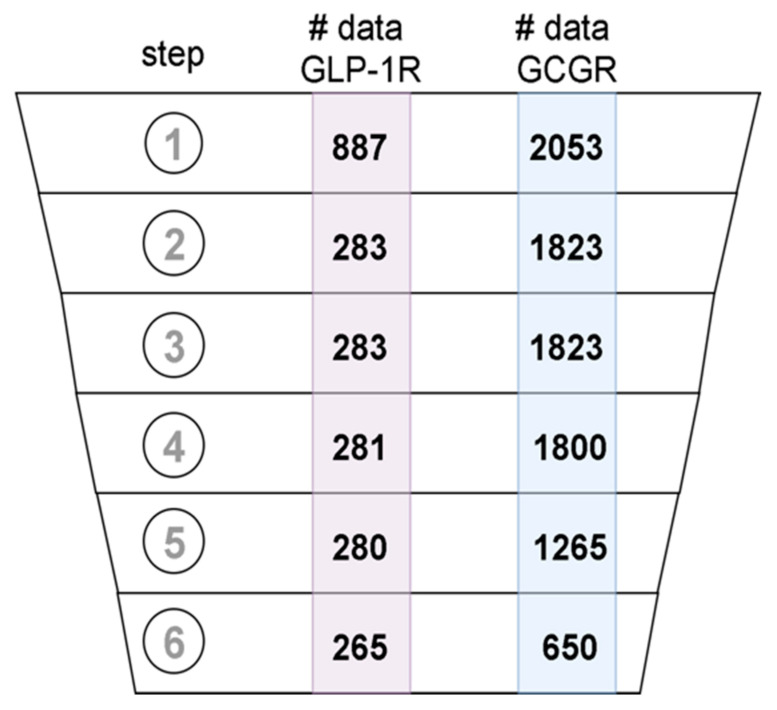
Data records that passed subsequent data curation steps: (1) data acquisition, (2) removing records with no SMILES included, (3) removing records with Confidence Score <8, (4) keeping only records from large assays, (5) removing records without numerical value for desired property, and (6) duplicates merging.

The datasets were curated following the best practices protocols [42]. What is more, we extended the existing guidelines to use ChEMBL-specific metadata in the curation pipeline. In Figure 9, we presented the number of ligands that were in the datasets after each step of data curation, starting from the initial dataset. In the first step, the ligand structures encoded in the SMILES format were converted to 2D molecular structure and standardized using the MolVS library (https://molvs.readthedocs.io/en/latest/) (accessed on: September 2020). Records that did not contain structural information were removed (see Figure 9). We used the confidence score reported in the assay metadata to assess the reliability of data. We included only data with a confidence score greater than 7 (see Figure 9). To further refine the selection, we choose the data produced only in large assays, i.e., ones that involved testing of more than 5 different compounds. Records that did not contain any numerical data about the desired property, e.g., EC_50_ for GLP-1R and IC_50_ for GCGR were removed. Duplicated structures in datasets were detected by comparison of their InChI identifiers. The duplicated entries were clustered and analyzed. Clusters containing inconsistent data, e.g., clusters of data that demonstrated significant standard deviation of experimental values, were removed from the datasets. The final curated datasets included: 650 compounds for GCGR and 235 compounds for GLP-1R. The number of compounds for the GIPR receptor (data not shown) was too small (107 compounds including 23 compounds similar to the binding site 4 PDB ligand), so we discarded this dataset from the current study. Vectors encoding Morgan fingerprints (2048-bit) with a radius of 3 were calculated for each chemical structure in the datasets.

### 4.4. Data Storage

Results of the current study have been added to the recently published repository of GPCR data: https://db-gpcr.chem.uw.edu.pl [27]. This repository includes annotated compounds of GLP-1R and GCGR receptors (compounds similar to the binding site 1 ligand and compounds similar to the binding site 4 ligand, respectively). Data on observed biological responses (pEC_50_ and pIC_50_) were also added for each compound. Structures and half-maximal response concentrations were visualized on the website with SMViewer.

### 4.5. Data Usage

#### 4.5.1. QSAR Modeling and Validation

QSAR modeling included the following stages: acquisition of data on EC_50_ and IC_50_ for GLP-1R and GCGR, respectively; data curation; Morgan fingerprints calculations; model development and validation. Morgan fingerprints were used as a feature vector for the machine learning-based development of the QSAR models for GCGR and GLP-1R. The machine learning algorithm used for QSAR modeling was Light Gradient Boosting Machines (LightGBM) [43]. A grid search was performed to select the best values for hyperparameters: the number of trees in an ensemble, regularization, and feature subsample fraction. The resulting model consisted of 100 boosted trees, feature subsampling was set to 0.1, and regularization to 1. The models were validated using a 5-fold cross-validation procedure. The Q^2^ validation scores were computed on five randomly selected nonintersecting subsets of data. For each test subset, the rest of the data was used to train the model. The predictions from each testing subset were used to calculate the Q^2^ score against the ground truth. The procedure was repeated 10 times, and the average Q^2^ was reported along with the standard deviation.

In detail, in 5-fold cross-validation, we divided the dataset into 5 non-overlapping test sets. Each test set consisted of ca. 20% of data, while the rest of the data of each test set were used as training examples. The model was independently trained and tested for each cross-validation data fold. The score calculated for each fold was averaged. The procedure was repeated 20 times to account for random effects that may affect averaged score due to different random splits of the test folds.

#### 4.5.2. Molecular Docking

Structures of transmembrane domains of GCGR and GLP-1R receptors were prepared in Maestro [41]. A maximum 10 residues in the binding site area were selected as flexible during molecular docking due to computational time (see Supplementary Table S1). The selected residues were in contact with PDB ligands (see Supplementary Table S1). Ligands were extracted as 2D sdf files from the ChEMBL datasets and then converted to 3D with OpenBabel [44]. Partial charges were computed in two ways: OPLS-AA [41,45] and Gasteiger [46] (MGL Tools 1.5.6). The former charges assignment provided a better correlation with pEC_50_/pIC_50_ experimental values (data not shown). Fully flexible (ligand and receptor) molecular docking was performed with Autodock VINA [34]. As a principle, no human intervention was applied to the above procedure, and therefore 5 and 8 compounds from GLP-1R and GCGR datasets, respectively, failed at the stage of the 2D to 3D conversion, the partial charges assignment, and/or molecular docking, so they were discarded from the molecular docking part of the study. Failed compounds contained, e.g., rediocide with a macrocyclic ring and 1,4-naphthyridine derivatives such as 6,7-dichloro-, 6-chloro-7-nitro-, 2-methylsulfonyl-, or 2-methylsulfinyl-1,4-naphthyridine. In each compound of the latter group, either S^δ+^O^δ−^ (sulfinyl) or N^δ+^O^δ−^ (from a nitro group) were present. Values of the Autodock VINA scoring function were juxtaposed with pEC50/pIC50 experimental results (see Section 2).

### 4.6. Statistical Analysis

The predictive performance of the model was characterized statistically with the Q^2^ metric calculated on held-out data for each fold from the cross-validation procedure. The metric was defined as: $Q^{2}=1-\frac{\sum {\left(y-\hat{y}\right)}^{2}}{\sum {\left(y-\bar{y}\right)}^{2}}$, where *y* is a vector of experimental target values, $\hat{y}$ is a vector of predicted target values on held-out data, and $\bar{y}$ is mean of experimental target values. A standard deviation of metrics within cross-validation folds was calculated to estimate the error of the predictive performance estimation. The predictive performance was reported as an average over cross-validation folds.

## Figures and Tables

**Table 1:** Diversity of small-molecule, non-peptide ligands binding to GLP-1R and GCGR receptors.

Receptor	PDB Id ^1^	Ligand	Ligand Type	Binding Site
GCGR	5XEZ, 5XF1 [6]	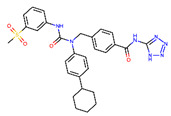	NAM ^2^	Binding site 4, allosteric, lipids-facing, outside of TMD ^3^
GLP-1R	5VEX [5]	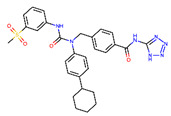	NAM	Binding site 4, allosteric, lipids-facing, outside of TMD
	5VEW [5,7]	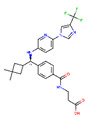	NAM	Binding site 4, allosteric, lipids-facing, outside of TMD
	6ORV [13]	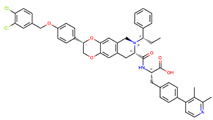	PAM ^4^	Binding site 1, allosteric, close to orthosteric ^5^
	6VCB [15]	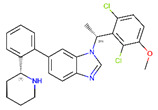	PAM	Binding site 3, allosteric, close to orthosteric
	7C2E [14]	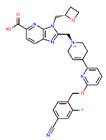	Full agonist	Binding site 2, orthosteric
	6X1A [17]	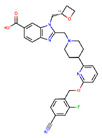	Full agonist	Binding site 2, orthosteric
	6XOX [16]	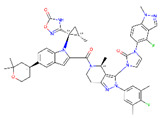	Full agonist	Binding site 2, orthosteric
	6X19 [17]	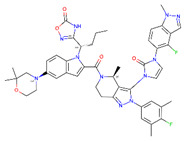	Full agonist	Binding site 2, orthosteric

^1^ PDB id—a Protein Data Bank identifier, ^2^ NAM—negative allosteric modulator, ^3^ The extra-helical binding site on the outside of TMD, located between TMH6 and TMH7, and facing the membrane, ^4^ PAM—positive allosteric modulator, ^5^ The binding site in the extracellular region of TMD, close to the orthosteric site.

## Data Availability

The data presented in this study are openly available on: https://db-gpcr.chem.uw.edu.pl/.

## Supplementary Materials

The following are available online at https://www.mdpi.com/article/10.3390/ijms22084060/s1.
