# Supplementary material for: Ligand-Receptor Interactions and Machine Learning in GCGR and GLP-1R Drug Discovery
Source: Int J Mol Sci. 2021 Apr 14;22(8):4060. doi: 10.3390/ijms22084060 (PMC8071054; doi:10.3390/ijms22084060)
Supplement: Supplementary file 1 [file ijms-22-04060-s001.pdf]

# Supplemental data

## Ligand-receptor interactions in machine learning-assisted GCGR and GLP-1R drug discovery

Mikołaj Mizera, Dorota Latek
Faculty of Chemistry, University of Warsaw
Pasteura 1, 02-093 Warsaw, Poland

This file includes Table S1 and Figures S1-S7 and that corresponds to the main manuscript text.

## Tables

**Table S1**. Flexible residues (grey) selected in binding sites 1-4 of GLP-1R and in the binding site 4 of GCGR with PDB ligands (yellow).

| Receptor | Binding site | PDB id | Residues kept as flexible during molecular docking |
|---|---|---|---|
| GLP-1R | 1 | 6ORV | 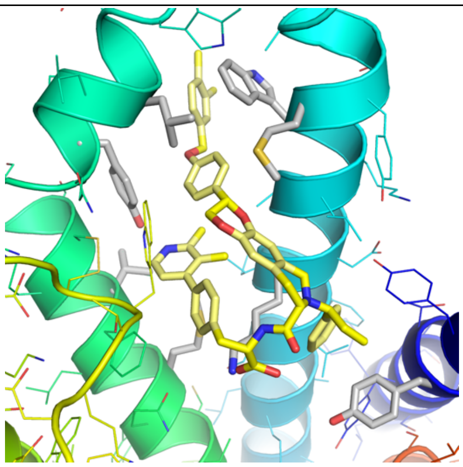 |
| | 2 | 7C2E | 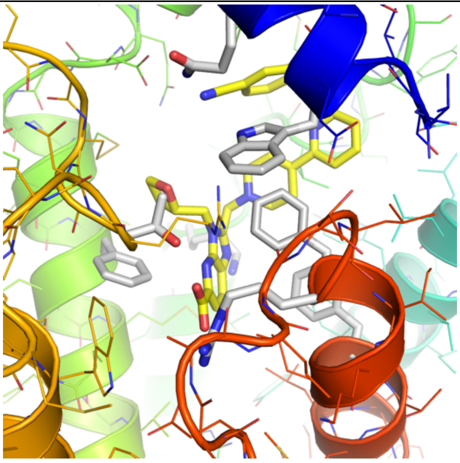 |

| | | |
|---|---|---|
| 3 | 6VCB | 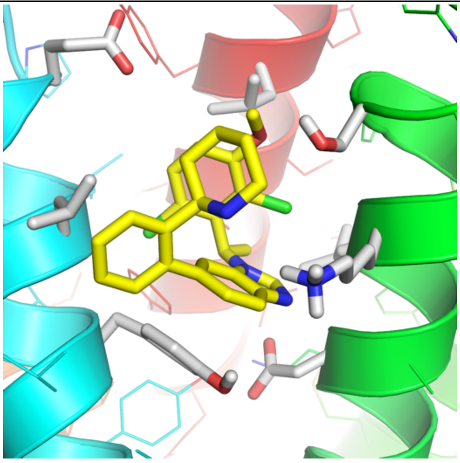 |
| 4 (I) | 6LN2 | 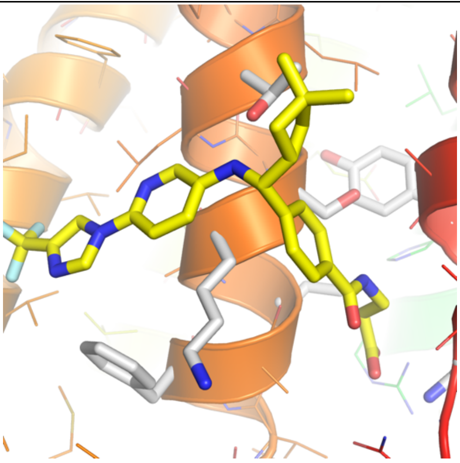 |
| 4 (II) | 5VEW | 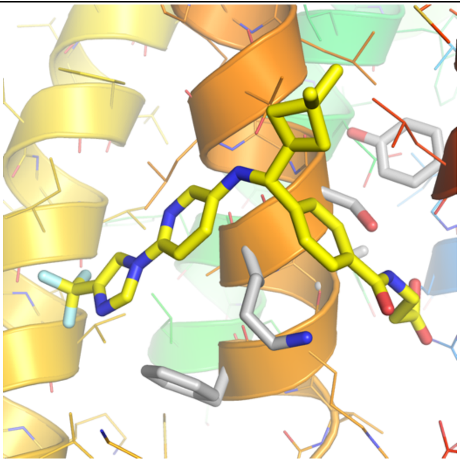 |

| | | | |
|---|---|---|---|
| GCGR | 4 | 5XEZ | 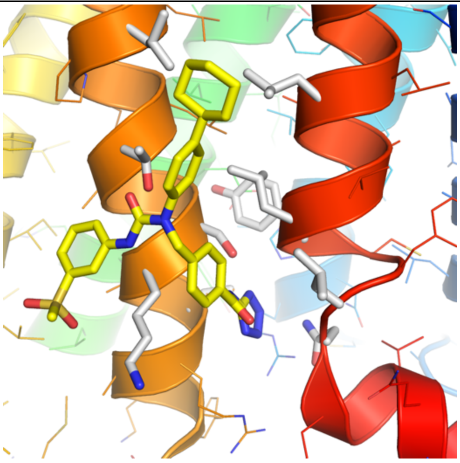 |

# Figures

**Figure S1**. Distribution of ChEMBL datasets – Daylight/Tanimoto coefficients vs. pEC50 (GLP-1R – left) and pIC50 (GCGR - right). GLP-1R dataset splits into two groups – one dissimilar to the site 1 ligands and another – similar to the site 1 ligands. The latter group demonstrates high pEC50. In the case of GCGR, ligands formed four clusters with decreasing number of members. Ligands similar to the binding site 4 ligand demonstrated rather high pIC50, though not the best possible.

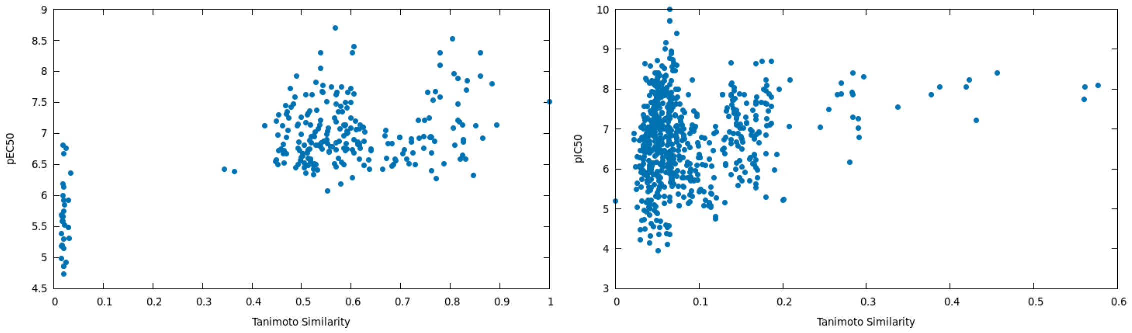


**Figure S2**. AlogP of the GLP-1R ligand set vs. Autodock VINA binding energy. Here, the binding site 1 from 6ORV was used in molecular docking. Autodock VINA scores (ligand binding energies) reflected the compound lipophilicity. Site 1 of GLP-1R demonstrated high affinity for lipophilic ligands.

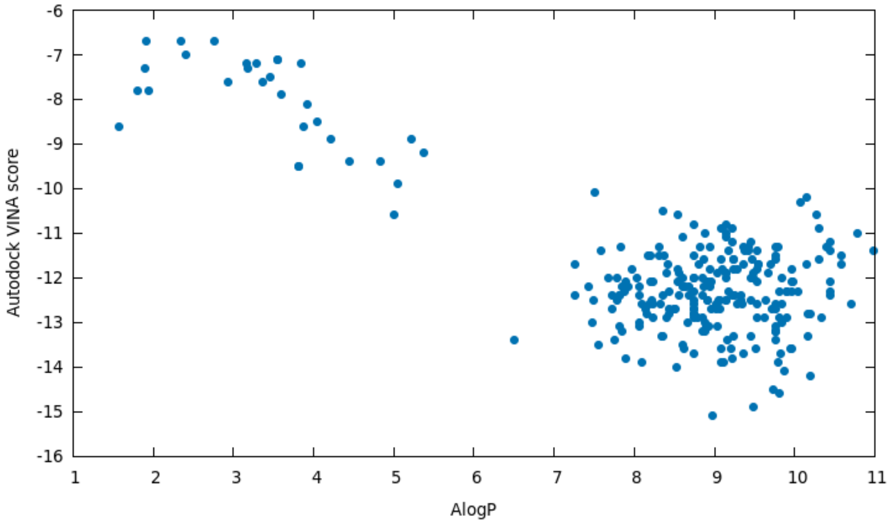


**Figure S3**. Results of docking of GLP-1R actives to all possible binding sites. Compounds similar to the binding site 1 ligand were scored better by Autodock VINA than the ‘other’ compounds in all cases. Results for docking to the binding site 1 are presented in the main manuscript. Inverse correlation coefficients were in the range of 0.4-0.6.

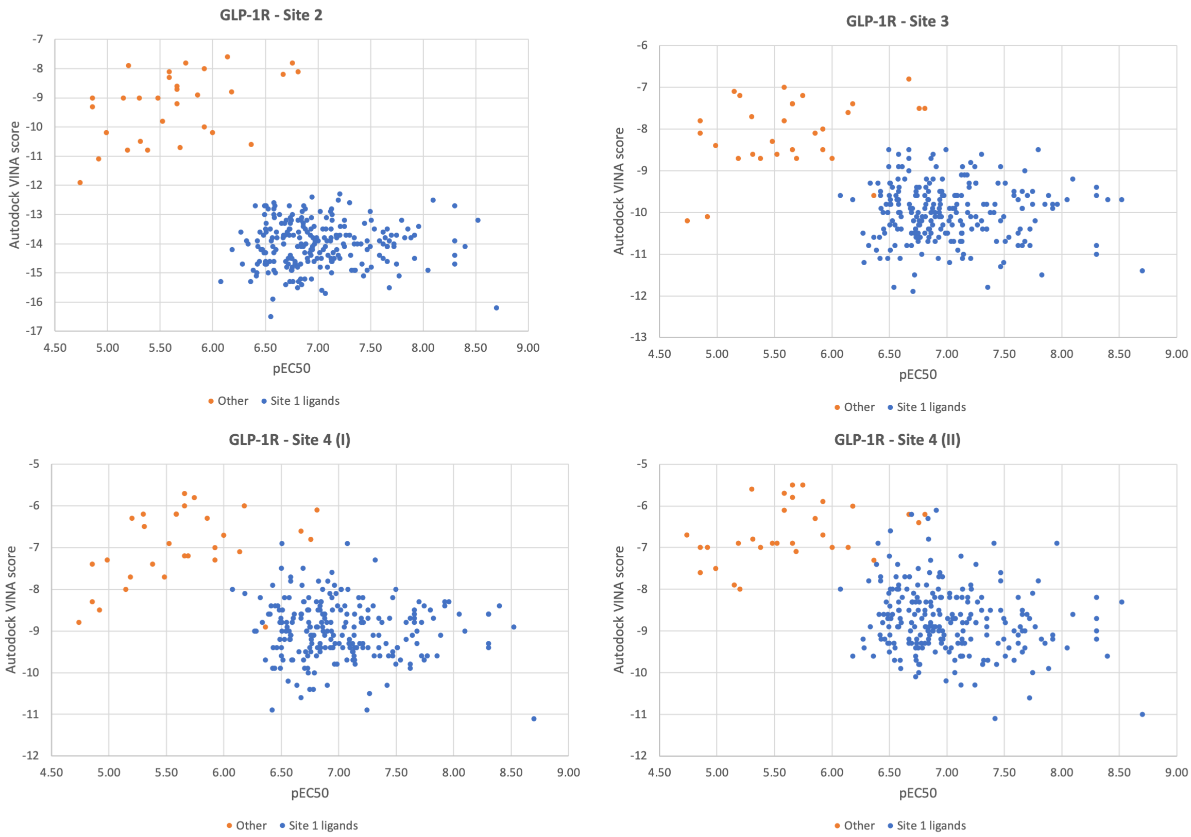


**Figure S4**. Autocorrelation plots of true vs. predicted values of EC50 (GLP1R) and true vs. predicted values of IC50 (GCGR).

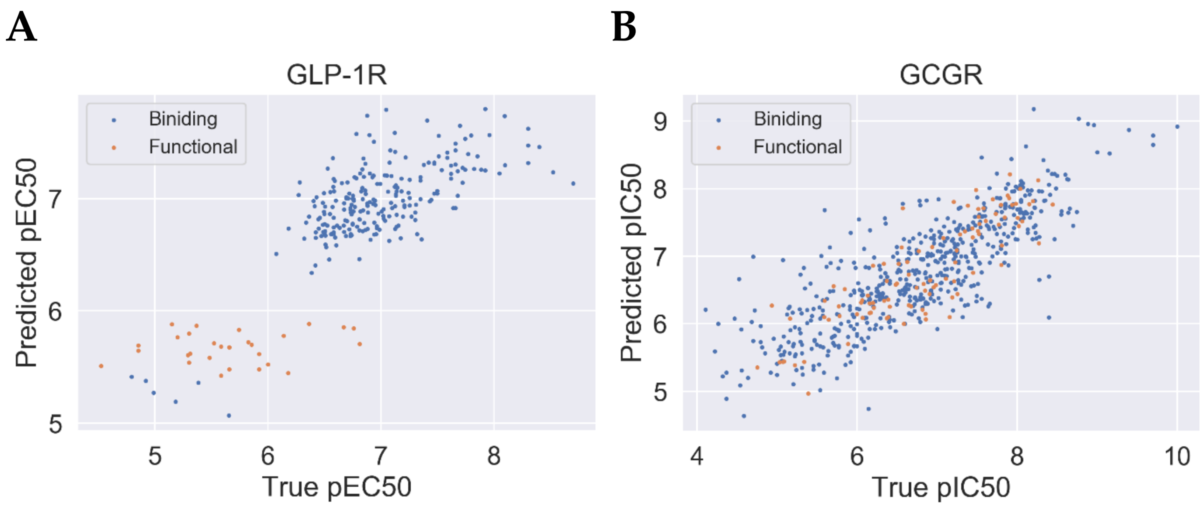


**Figure S5**. Multidimensional scaling embedding (MDS) for the relative similarity of ligand structures in GLP-1R dataset (A) and GCGR dataset (B). 'True pEC50/IC50' means in this case 'Experimental pEC50/pIC50'.

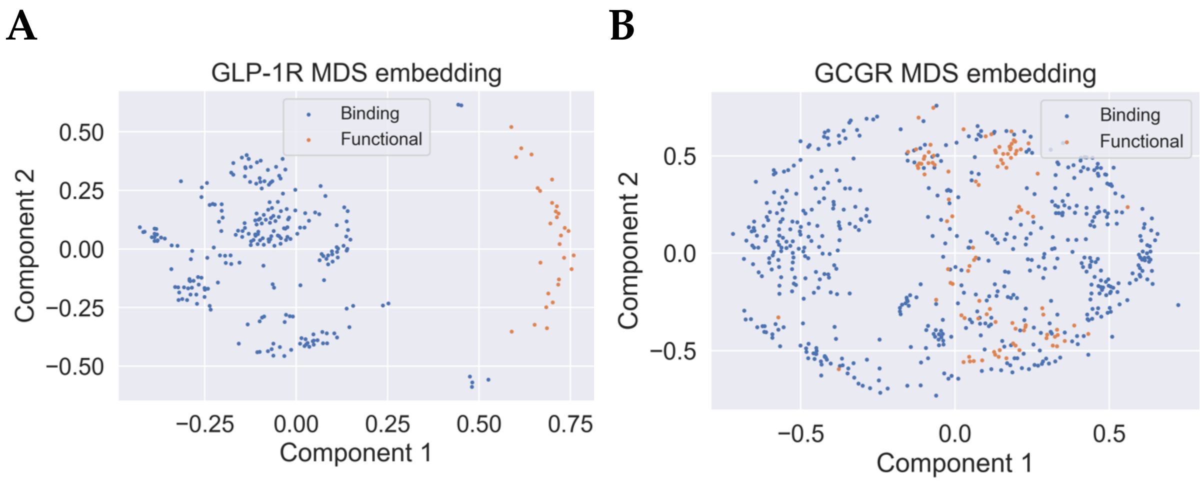


**Figure S6**. Compounds derived from the CMAUP database with confirmed activity for GCGR. Although both compounds are dissimilar to any of PDB ligands, compound NPC471603 (left) from *Mammea siamensis* could be modified to fit the V-shape of known NAMs. Molecular weight and shape of compound NPC62792 (right) from *Trigonostemon reidioides* - suggest that it would rather bind to the orthosteric site, if to any.

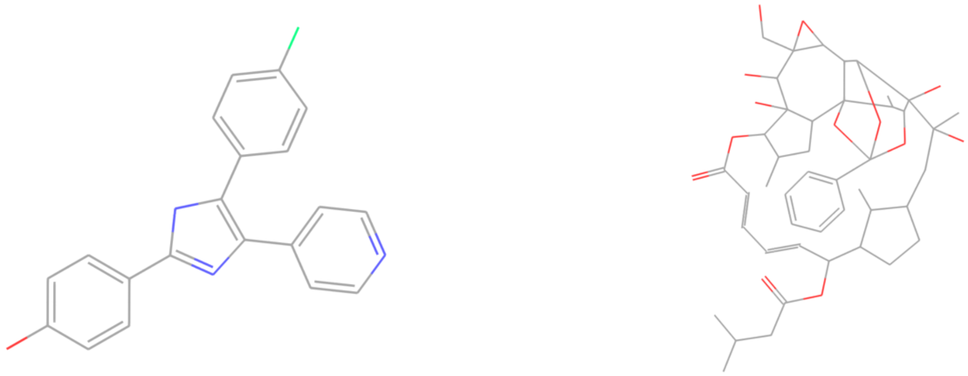

**Figure S7**. A distinct compound found with our model in *Mammea siamensis* extract for GLP-1R. This compound is not similar to any of GLP-1R actives that bind to sites 1-4.

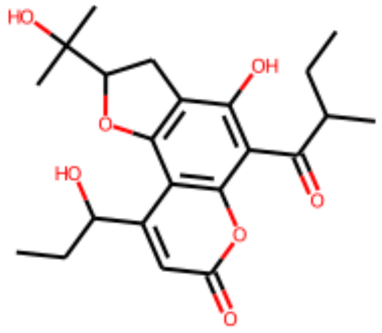


NPC470908
